# Animal Models of Maladaptive Traits: Disorders in Sensorimotor Gating and Attentional Quantifiable Responses as Possible Endophenotypes

**DOI:** 10.3389/fpsyg.2016.00206

**Published:** 2016-02-19

**Authors:** Juan P. Vargas, Estrella Díaz, Manuel Portavella, Juan C. López

**Affiliations:** Animal Behavior and Neuroscience Lab, Department of Experimental Psychology, Universidad de SevillaSeville, Spain

**Keywords:** dopamine, endophenotype, latent inhibition, mental disorder, prepulse inhibition

## Abstract

Traditional diagnostic scales are based on a number of symptoms to evaluate and classify mental diseases. In many cases, this process becomes subjective, since the patient must calibrate the magnitude of his/her symptoms and therefore the severity of his/her disorder. A completely different approach is based on the study of the more vulnerable traits of cognitive disorders. In this regard, animal models of mental illness could be a useful tool to characterize indicators of possible cognitive dysfunctions in humans. Specifically, several cognitive disorders such as schizophrenia involve a dysfunction in the mesocorticolimbic dopaminergic system during development. These variations in dopamine levels or dopamine receptor sensibility correlate with many behavioral disturbances. These behaviors may be included in a specific phenotype and may be analyzed under controlled conditions in the laboratory. The present study provides an introductory overview of different quantitative traits that could be used as a possible risk indicator for different mental disorders, helping to define a specific endophenotype. Specifically, we examine different experimental procedures to measure impaired response in attention linked to sensorimotor gating as a possible personality trait involved in maladaptive behaviors.

## Introduction

The criteria used by current diagnostic scales are based on the analysis of external symptoms of the patient. Disorders such as attention deficit with hyperactivity or mental disorders such as schizophrenia are diagnosed based on symptoms that, in many cases, require the patient to evaluate their intensity. This situation creates a serious problem for the diagnosis, given the large amount of subjective information handled by the psychologist or the psychiatrist (Robbins et al., 2012).

The problem of subjectivity and comorbidity in diagnostic errors are, in part, a consequence of the absence of biological markers to facilitate proper classification of the disorder. With relative ease, the diagnostic manuals such as the DSM or ICD propose a continuous change in the criteria for inclusion or exclusion of a disorder due largely to the heterogeneity and complexity of symptoms that define that disorder. These are so complex that patients with different symptoms might have the same diagnosis, a fact that significantly increases the difficulty of providing proper treatment. This high comorbidity between various diseases indicates a clear deficiency in the classification system of mental disorders, preventing the identification of valid pathologies (Hyman, 2010). It is possible that the psychotherapeutic and pharmacological failures are largely due to this fact. Note for example that the therapeutic effectiveness of pharmacological treatments reaches approximately 50% (Wong et al., 2010).

Using a diverse group of pharmacological treatments to relieve disorders such as depression is also an indicator of the disparity of its diagnosis. For example, the use of inhibitors of serotonin reuptake is applied for a specific type of depressive symptoms, which differs from those used under MAO inhibitors or under tricyclics. The differential response of each patient to treatment indicates that disorders included in the same category should be treated with different principles. Alternatively, this phenomenon could be indirectly indicating that different types of disorders within a category may have a different biological basis.

An alternative to this traditional view is the characterisation of endophenotypes. An endophenotype is a quantitative measurable trait associated with a genetic predisposition (Gottesman and Shields, 1972, 1973). In contrast to the symptomatic view of psychopathology, the endophenotype analyses the characteristics that show possible brain vulnerability to suffer a specific type of disorder. The objective is the study and quantification of specific features that reflect a mental disorder associated with a biochemical sign (Hasler et al., 2006; Turetsky et al., 2007). Throughout its long history, the functional study of behavior in the laboratory has provided a number of indicators that could serve as markers for selective expression of the maladaptive behaviors. Applying this model to the field of psychopathology, mental disorders could be considered as extremes at one or both tails of these normal distributions (Miller and Rockstroh, 2013). From this point of view, psychopathology would view disorders as dimensional notions, and not as categories under a binary diagnosis (Hyman, 2010; Frances and Widiger, 2012; Morris and Cuthbert, 2012).

Here, we provide a set of measurable procedures sensitive enough to be used to identify possible endophenotypes developed from animal models. These endophenotypes are based on the correlation between brain processes and measurable responses of a subject that enable us to discriminate between different sets of symptoms, and facilitate new specific therapies. In addition, the evaluation of these traits could facilitate a more objective classification system of psychopathologies.

## How does the use of an Animal Model Contribute to Psychopathology Classification?

The recent developments in genetics and epigenetics allow us to better approach understanding behavior and facilitate the understanding of mental disorders. The fact that some behaviors have a Mendelian basis, suggests the possibility of finding simple mutations that affect behavior in a relatively specific manner. However, there are only a small group of features known as Mendelian traits (or traits 1:1) in relation to genotype. Mental disorders such as depression or schizophrenia are clearly polygenic, or may also be generated by various mutant alleles of the same gene and specific environmental conditions, making the analysis of their causes a complex procedure (Zahn-Waxler et al., 1988; Winokur and Kadrmas, 1989; Kidd, 1997; Moldin, 1997; Owen, 2000; Torrey and Yolken, 2000; Goldman, 2012). Moreover, these illnesses are the result of the interactions of both genetic and epigenetic factors. And although we now have suitable tools for genotype analysis, the fact that these etiological factors -genes and environment- interact to produce similar phenotypes, significantly increases the difficulty to precisely define the specific weight of each one in the generation of behavior (Plomin and Rende, 1991). Identifying what groups of genes may contribute to the expression of a disorder is a long process of molecular genetics. However, the identification of relating groups of genes with specific traits is currently a more achievable goal.

The use of animal models for the study of personality traits, vulnerability to certain disorders or substance abuse dependence is an interesting strategy for developing behavioral protocols in the laboratory. Although in some cases these models could show poor face and predictive validity, the construct validity associated with the etiology or mechanism of the underlying disorder is usually high (O’Donnell, 2011). For example, animal models of schizophrenia have been successful in evaluating risk factors (see **Table 1**). This fact is crucial in order to develop new pharmacological treatments or genetic therapies. However, the reduced face validity is often a problem when applying to human models.

**Table 1:** Several animal models have studied schizophrenia.

		MAM	NVHL
Executive functions	Attentional processes	Flagstad et al., 2005; Featherstone et al., 2007	
	Working memory deficits	Flagstad et al., 2005; Hazane et al., 2009	Chambers et al., 1996; Lipska et al., 2002
	Perseveration	Moore et al., 2006; Hazane et al., 2009	Marquis et al., 2008
	Recognition deficits	Featherstone et al., 2007	Sams-Dodd et al., 1997; Bachevalier et al., 1999
Motivational behavior	Increased liability for addictive behaviors	Flagstad et al., 2005	Swerdlow et al., 2001; Brady et al., 2008
	Responses to stress	Le Pen et al., 2006; Hazane et al., 2009	Sams-Dodd et al., 1997
Activity	Hyperlocomotion	Le Pen et al., 2006; Moore et al., 2006; Penschuck et al., 2006; Hazane et al., 2009	Lipska et al., 1993; Wan et al., 1996
Information filtering mechanism	Sensorimotor gating deficits	Le Pen et al., 2006; Moore et al., 2006; Hazane et al., 2009	Swerdlow et al., 1995

*Pharmacological models have used amphetamine, PCP or NMDA to simulate some of the symptoms. However, only two models have shown the illness as a developmental process. The neonatal ventral hippocampal lesion (NVHL) and MAM models showed marked maladaptive behavior when animals reached adulthood. Le Pen et al. (2011) and O’Donnell (2011) have described several behavioral procedures where we can find similar results with different techniques aimed at developing a dysfunctional PfC.*

The development of endophenotypes is one alternative to try to improve this model. Taking advantage of high construct validity, we can develop sensitive tests for quantifying specific traits. Measures such as latent inhibition (LI) or prepulse inhibition (PPI) are, among others, easily quantifiable under controlled conditions in the laboratory. In addition, we can use the advantage of these procedures in a similar way in both animals and humans, and the results are easily extrapolated from an animal model to a human model (Le Pen et al., 2011). While PPI is a very simple procedure seeking to analyze early attentional gating mechanisms, the LI is a learning process related to selective attention and habituation to irrelevant information (Lubow and Gewirtz, 1995; Swerdlow et al., 1996; Braff and Swerdlow, 1997). Animal models indicate that problems in the expression of PPI or LI correlate with cognitive deficits such as working memory or alternation behavior, locomotion activity such as hyperactivity induced by a dopamine receptor agonist, and some negative symptoms also described in pathologies such as schizophrenia (Flagstad et al., 2004; Le Pen et al., 2006; Moore et al., 2006; Hazane et al., 2009). For example, patients with schizophrenia show these symptoms associated with a dysfunctional prefrontal cortex (PfC; Manoach, 2003; Silver et al., 2003; Godsil et al., 2013), therefore, a behavioral test aimed to evaluate PfC function is a useful tool for an accurate differential diagnostic. The knowledge acquired in recent years on the use of a quantifiable measurement of these traits is boosting the development of unified models of diagnosis that include data from all levels, that is: genetics, biochemical, and behavioral levels.

But the question is, how can we contribute to this proposal? Consider, for instance, one of the most complex disorders, schizophrenia. Currently, schizophrenia is an umbrella term for a diverse group of disorders with possibly different etiologies. Focusing on PfC dysfunction, animal research has provided explanatory models to understand the possible development of this mental disturbance (O’Donnell, 2011; Godsil et al., 2013). Procedures aimed to alter gestation and fetal development such as the MAM model (Methylazoxymethanol), or techniques affecting the maturation process of PfC such as ventral hippocampus lesion in neonates, allow us to experimentally analyze this disorder (Waddington et al., 1999; Bramon et al., 2005; Chambers and Lipska, 2011). Both procedures show a clear PfC dysfunction (Tseng and O’Donnell, 2004, 2007). Cells unit recording studies indicate the possibility of a deficit in inhibitory GABAergic cells. This could be the cause of an excessive release of dopamine cells in the mesocortical system (Tseng and O’Donnell, 2004, 2007; O’Donnell, 2011; Godsil et al., 2013). This could be the reason that PPI or LI could be affected in these animal models and in schizophrenia. Both behavioral processes require an operative PfC for a normal expression. PPI and LI are very sensitive to disturbances in this structure. In this regard, a deficit in one or both processes could be a risk factor. On the whole, the characteristics of a dysfunctional PfC and the impairment in LI and PPI expression could be signs of a specific type of mental disorder, apart from the current model of mental illness where the disorder and its severity are expressed in terms of a scale filled out by the patient or a close family member.

## Dopaminergic System as a Sign of a Possible Risk Factor

The function of the dopamine neurotransmitter has attracted great interest because of its relationship with the processes of learning and with several mental disorders such as schizophrenia, depression, ADHT or addiction to a substance of abuse (Robbins, 1992; Feldman et al., 1997; Weiner, 2003; Grace and Sesack, 2010; Simpson et al., 2010; Wise, 2010; Milad and Rauch, 2012; Díaz et al., 2015). The distribution of dopaminergic neurons is abundant in the central nervous system. The midbrain neurons and their efferences to the ventral striatum and PfC play a special role in the learning process (Robbins and Everitt, 1996). Dopaminergic pathways of the ventral tegmental area (VTA) toward the nucleus accumbens (NAc) are closely linked to the motivational processes of learning (Berridge and Robinson, 1998; Berridge, 2007). Many stimulant drugs, such as cocaine or amphetamine, operate in this place, and their function significantly increases the release or reduces the reuptake of dopamine in the system.

Dopamine receptors belong to the G-protein coupled receptors family. All these receptors possess seven transmembrane domains and five subtypes of dopamine receptors according to their molecular characteristics. These have been grouped into two pharmacological families according to the effect produced by agonists and antagonists. D1 family includes the subtypes D1 and D5 receptors. Both stimulate adenylyl cyclase, producing cAMP. On the other hand, D2 receptor family includes the subtypes D2, D3, and D4. These receptors inhibit the formation of cAMP. The D1 receptor is the most abundant in the central nervous system (Missale et al., 1998). The greatest concentration of this receptor is found in the neostriatum, NAc, amygdala, and substantia nigra. However, its affinity for dopamine is relatively low. The D2 receptor is found in high concentrations in the neostriatum (GABAergic neurons) in the NAc and hippocampus, and with a moderate density in the substantia nigra, cerebral cortex, globus pallidus, thalamus, and hypothalamus. These data make D1 and D2 receptors specific targets for the study of cognitive, emotional, and motivational disorders. Electrophysiological studies have made important contributions concerning their functional activity in the mesolimbic system (O’Donnell and Grace, 1998; Moore et al., 1999; Grace, 2000; Floresco et al., 2001; Goto and Grace, 2008). These studies are of great relevance given the importance of these receptors in the processes of associative learning. Recent studies have shown that the D2 receptor is located in the projections of both the PfC and the amygdala in the form of autoreceptors (O’Donnell and Grace, 1995; Groenewegen et al., 1999; Goto and Grace, 2008). Specifically, D2 receptors are located in the presynaptic areas with the function of modulating the dopaminergic activity of the VTA over NAc through excitatory projections. That is why this receptor has been linked to the goal directed processes or controlled processes that require high attentional activity (O’Donnell and Grace, 1995; Goto and Grace, 2008). In contrast, the activity of D1 receptors in the mesolimbic system is different than the one described for D2. These are located in the post-synaptic cells of the NAc that receive glutamatergic afferences from the hippocampus and dopaminergic afferences from the VTA (O’Donnell and Grace, 1995; Groenewegen et al., 1999; Goto and Grace, 2008).

Disturbances in this system increase the risk of developing serious mental illness (O’Donnell, 2011; Godsil et al., 2013). Disorders such as schizophrenia have been linked directly to disturbances during brain development associated with the second trimester of pregnancy (Waddington et al., 1999; Bramon et al., 2005). Changes in the dopaminergic sensitivity and in the levels of dopamine or dopamine receptors volume could be the result of this process. Specifically, the family of D2 receptors seems to be more related to the disease process (Grace and Sesack, 2010; Simpson et al., 2010; Wise, 2010; Milad and Rauch, 2012), since the antagonists of these receptors such as haloperidol are effective in reducing symptoms (Lubow and Weiner, 2010). This is the reason why it was suggested a substantial increase of this type of receptors underlies this disorder as shown, for instance, in the studies of post-mortem tissue (Seeman and Nizkik, 1990).

Currently the drug treatment of disorders such as schizophrenia or ADHT act directly on the dopaminergic modulation in the brain. The changes that cause the blockade or stimulation of receptors of this neurotransmitter can be studied in the laboratory. Changes in the sensorimotor gating or selection of relevant stimulus of the environment can be considered as possible quantifiable traits directly related with the level of dopamine or dopaminergic receptors in the mesocortical and mesolimbic system. It is important to emphasize that injuries to PfC produce dopamine dysregulation and deficits in PPI and LI expression.

## PPI of Startle Response. A Sensorimotor Gating Measure

The startle response to an intense stimulus is a reflex behavior that has been described in all mammals studied. This is a fast-twitch of the skeletal muscle that leads to processing environmental stimuli and guiding the attention of the subject to a possible threat. This type of response is interesting because it has been associated with specific genes that appear in schizophrenia and as a possible trait with endophenotypical characteristics. For example, Vaidyanathan et al. (2014) studied the startle blink reflex using a very large human sample. Analyzing the startle response, they found a heritable specific pattern of behavior in the sample. In addition, this trait was associated with candidate genes in the endophenotype of schizophrenia. However, although it is an automatic reaction, the outcome can be modulated by the previous presence of a stimulus of lower intensity, therefore PPI is defined as the attenuation of the startle response to an intense pulse when it is preceded by a lower-intensity prepulse stimulus. When the prepulse is perceived, the mechanism of startle is inhibited and the animal displays a lower response (Graham, 1975; Lüthy et al., 2003; Larrauri and Schmajuk, 2006).

The problems with sensorimotor gating have been linked with the levels of dopamine in the NAc. The NAc integrates information from different structures, and even though dopamine modulation in NAc is dependent on mesocortical and mesolimbic systems (Ellenbroek et al., 1996; Larrauri and Schmajuk, 2006), the selective modulation of PfC afferent transmission is especially relevant. PfC afferences could facilitate behaviors oriented to specific goals, and a dopamine deficit could be involved in the incapacity to control the behavior (Goto and Grace, 2008).

It should be noted that the dopaminergic innervation of the PfC increases progressively through adolescence until adulthood. In this period, we can find modifications in density, shape and organization of the circuits (Kalsbeek et al., 1988; Benes et al., 2000; Seamans and Yang, 2004; Segalowitz and Davies, 2004; Manitt et al., 2011; Naneix et al., 2012). A mature circuit allows the dopaminergic neurons to fit their responses in an adaptive way, modulating their response in correlation with environmental changes (Spear, 2000; Tseng and O’Donnell, 2004, 2007; O’Donnell, 2011; Cass et al., 2013; Godsil et al., 2013). Currently, it is estimated that delays or alterations in the maturation process of the PfC dopamine system could be the cause of a large number of mental disorders (O’Donnell, 2011; Godsil et al., 2013). Specifically, a poor inhibitory capacity of the PfC over the NAc may be the major etiological factor in severe disorders such as schizophrenia. In fact, a deficit in the response to the pulse has been observed in different types of cognitive disorders, and it is specifically relevant in patients with schizophrenia (Braff et al., 1992, 2001a,b). Thus, a reduced PPI could be used as a trait for attentional deficit, besides being included as a schizotypy personality trait or a possible endophenotype of schizophrenia (Cadenhead et al., 2000; Braff, 2010; O’Donnell, 2011).

However, this trait is not specific for patients with schizophrenia but indicates a trait of vulnerability, and it is very clear in patients with schizophrenia. In this regard, the PPI deficit could be a necessary condition as a risk factor of schizophrenia, but it could not be sufficient by itself. The PPI deficit might be found in several disorders, and a pathological process such as schizophrenia needs other indicators.

## PPI, Dopamine and Impulsivity: A Trait, A Neurotransmitter and A Quantifiable Measure not Associated Exclusively With Schizophrenia

PPI is an easy system to measure in animals including humans. It has been used in animal models of schizophrenia, even though there are several studies where this procedure has been correlated with impulsivity traits. López et al. (2015) analyzed the PPI in rats classified as impulsive by an autoshaping procedure. Animals designated as sign trackers showed approach behavior to a conditional stimulus before delivery of unconditional stimulus. Specifically, for sign tracker animals (STa) the conditional stimulus could be a surrogate of the unconditional stimulus (Flagel et al., 2007; Robinson and Flagel, 2009). These kind of animals showed high levels of dopamine in NAc, but only in the presence of a conditional stimulus (Flagel et al., 2011). These data were consistent with the results of López et al. (2015) using a PPI procedure. In fact, the STa showed a lower PPI response to stimuli of low intensity. This reduced inhibitory ability of the STa showed a difference in the behavioral pattern in normal animals. Furthermore, these data may indicate that ST subjects may be more vulnerable to cognitive disorders in which dopamine is involved.

An important question about the vulnerability of STa to an impulsive behavior comes from specific activity of D2 subtype dopamine receptor. This receptor is located presynaptically on PfC terminals, and has been related with a selective modulation of the NAc to facilitate goal-directed behaviors (Goto and Grace, 2008). In addition, several psychopathologies associated with PfC have shown a deficit between this structure and the projections to NAc (O’Donnell, 2011). López et al. (2015) found a possible vulnerability from STa, since these animals showed a large sensibility of D2 receptor to the administration of an agonist such as quinpirole. This drug affected only STa performance, indicating that this type of trait differs from that observed in schizophrenia. It would be appropriate at this stage to point out the difference between an animal model of impulsivity and an animal model of schizophrenia regarding a dysfunction in PfC. These models have developed several protocols to evaluate attentional processes, and LI is a perfect candidate to discriminate between impulsivity and schizophrenia, because it allows for evaluating attention and executive functions, both specific to PfC function. Impulsive models of animals have found differences in incentive salience of the conditional stimulus, but not in attentional problems (Berridge and Robinson, 1998; Berridge, 2007) such as in schizophrenia models.

## LI, Dopamine and Attentional Deficits

LI is a learning process observed when the acquisition of a conditional response to a conditioned stimulus paired with a reinforcer is retarded if the same stimulus has previously been pre-exposed in the absence of the reinforcer. LI pharmacology has been associated almost exclusively with the use of an animal model of schizophrenia, and is therefore largely consistent with the pharmacology of schizophrenia (Lubow and Kaplan, 2010; Lubow and Weiner, 2010; Díaz et al., 2015). Specifically, because some of the symptoms of schizophrenia are characterized by an inability to filter, or ignore irrelevant or unimportant stimuli, an anomalous LI was proposed as a tool for the study of possible deficits of attention (Lubow and Weiner, 2010).

Again, dopaminergic activity of the NAc is the essential neural substrate for its expression. Animal models have shown that the primary role of the NAc is to restrict the expression of LI under certain conditions, and thus ensure that the LI is flexible and sensitive to environmental demands. It is important to highlight that, in the absence of modulator mechanisms responsible for restricting the expression of LI to the specific conditions, the effects of an irrelevant stimulus would be extremely robust and maladaptive. In this regard, LI might reflect the psychological processes that are impaired in schizophrenia, since most of the patients showed a reduced expression of this phenomenon. The identification of brain regions whose damage leads to disrupt the LI, joined with the studies of different parameters of expression in animal models, can provide important information on the dysfunctional brain circuits in schizophrenia. In previous decades it was suggested that some kind of hyperactivity of the dopaminergic systems represent a primary biochemical alteration in schizophrenia, which apparently constituted at least a plausible justification for biochemical alteration in this disorder (Iversen, 1976).

To gain insight into quantifiable attentional processes in LI, Díaz et al. (2014) analyzed the effect of various types of pre-exposure to a stimulus. The results indicated that there is a transfer from the ventral to the dorsal striatum in the processing of environmental information. In addition, the dorso-medial striatum is key to encode stimuli when these become irrelevant due to the lack of consequences after their presentation. A deficit in PfC could be the cause of a loss of transfer from ventral to dorsal striatum. Currently there are some laboratories working on this possibility. The inability to modulate dopamine in NAc does not allow for attentional disengagement, showing a persistent state of continuous attention.

The inability of encoding irrelevant information is one of the clearest deficits observed in patients with schizophrenia. Many modern learning theories assume that the amount of attention to a signal depends on how well the signal predicts the significant event of the past. Schizophrenia is associated with attention deficit and recent theories of psychosis have argued that positive symptoms such as delusions and hallucinations are related to a lack of selective attention. Patients with schizophrenia, who had severe positive symptoms, showed a clear difficulty in discriminating between predictive and non-predictive cues when compared to healthy adults. In addition, the rate of learning about non-predictive signals correlated with more severe positive symptoms in schizophrenia. These results suggest that the positive symptoms of schizophrenia were associated with increased attention, both to signals that are likely to be predictive and to those that are not predictive for causal learning. This selective attention deficit was the result of learning irrelevant causal associations (Morris et al., 2013). In this regard, the development of specific protocols to differentiate the expression of LI could be used as a possible risk factor in the population.

However, the complexity of this disorder suggests the possibility of different etiological factors may underlie the disease. At present there are many contradictory results regarding whether IL is affected in schizophrenia. Lubow and Kaplan (2010) addressed this issue in a recent review. They emphasize the difference between positive and negative symptoms in relation to the expression of IL. For instance, patients with high levels of negative symptoms and low of positive showed a potentiated LI. This data is relevant, because they could be observing different symptoms of the illness or different illness.

## Concluding Remarks

The mesocortical input of dopamine and the PfC play a critical role in normal cognitive processes and in several neuropsychiatric diseases. This dopamine input regulates aspects of working memory, planning and attention, among others. Similarly, some disturbances may be the basis for a variety of positive and negative symptoms, and therefore of many of the cognitive deficits associated with mental illness. Despite intensive research, we still have a lack of understanding of the basic principles of dopamine activity in the PfC and all the mesolimbic system. In recent years, there has been considerable effort to understand the cellular mechanisms of modulation of dopamine neurons in the PfC and its relationship with behavior. However, the results of these efforts have often led to contradictions and disputes (Nieoullon, 2002). Given the complexity of the function of the mesolimbic and the dopaminergic systems, the development of new tools will be necessary to facilitate discrimination of diagnostics and to provide a more objective assessment of the current classification systems. Namely, we suggest a shift or reconsideration in diagnostic scales adding other indicators. Clinical psychology has many tools to evaluate PfC dysfunction (for a review see Gruszka et al., 2010). We propose that PPI and LI could help to develop a new classification system, where we could distinguish between a psychotic illness such as schizophrenia by a dysfunction in PfC dopamine from other types of schizophrenia included in current scales. As we indicated above, current classification systems could be considering a diverse group of disorders under the same term of schizophrenia illness, and the different combination of positive and negative symptoms could indicate the severity of the disorder. The in depth analysis of these mechanisms, combined with genetic factors, is a new view that could facilitate the development of diagnostic categories in a more specific way and, therefore, a new therapeutic perspective in the future.

## Author Contributions

All authors contributed similarly in the theoretical development of the manuscript.

## Conflict of Interest Statement

The authors declare that the research was conducted in the absence of any commercial or financial relationships that could be construed as a potential conflict of interest.
